# Supplementary material for: The Antagonizing Role of Heme in the Antimalarial Function of Artemisinin: Elevating Intracellular Free Heme Negatively Impacts Artemisinin Activity in Plasmodium falciparum
Source: Molecules. 2022 Mar 8;27(6):1755. doi: 10.3390/molecules27061755 (PMC8949904; doi:10.3390/molecules27061755)
Supplement: Supplementary file 1 [file molecules-27-01755-s001.zip › molecules-1597551-supplementary.pdf]

molecules

Supplementary Information

The Antagonizing Role of Heme in the Antimalarial Function of Artemisinin: Elevating Intracellular Free Heme Negatively Impacts Artemisinin Activity in *Plasmodium falciparum*

Pan Zhu [1] and Bing Zhou [1,2,*]

[1] State Key Laboratory of Membrane Biology, School of Life Sciences, Tsinghua University, Beijing 100084, China
[2] Shenzhen Institute of Synthetic Biology, Shenzhen Institute of Advanced Technology, Chinese Academy of Sciences, Shenzhen 518055, China
* Correspondence: bing.zhou@siat.ac.cn

Contents

Supplementary Tables S1 and S2

Supplementary Figures S1–S6

Supplementary Tables

Supplementary Table S1. EC_{50} values of artemisinins with heme related chemicals.

Table S1A. Antimalarial effectiveness of artemisinins was reduced by exogenous hemin.

Sample			EC_{50} Value (nM)	*p*-Value (*t*-test)
Figure 1A	ART	Control	22.20 ± 1.13	/
		5 μM Hemin	90.59 ± 6.70	<0.0001 (***)
		10 μM Hemin	156.95 ± 10.35	<0.0001 (***)
Figure 1D	DHA	Control	12.52 ± 0.35	/
		5 μM Hemin	40.92 ± 1.58	<0.0001 (***)
		10 μM Hemin	77.48 ± 3.91	<0.0001 (***)

Table S1B. Hemozoin inhibitor-14c decreased sensitivity of artemisinin.

Sample			EC_{50} Value (nM)	*p*-Value (*t*-test)
Figure 2B	ART	Control	18.29 ± 0.26	/
		3 μM 14c	24.87 ± 0.68	<0.0001 (***)
		5 μM 14c	29.34 ± 1.82	0.0005 (***)
Figure 2C	DHA	Control	8.86 ± 0.57	/
		3 μM 14c	18.41 ± 2.46	0.0028 (**)
		5 μM 14c	30.92 ± 6.45	0.0041 (**)
Figure 2E	ART	Control	17.89 ± 0.52	/
		2 μM 14d	16.84 ± 0.64	0.0921 (ns)
		4 μM 14d	16.71 ± 0.83	0.1052 (ns)

Table S1C. HO neutralized 14c's effect on artemisinin sensitivity.

Sample			EC_{50} Value	*p*-Value (*t*-test)
Figure 3B<br>Supplementary Figure 4A	ART	3D7	25.95 ± 1.45 nM	/
		HO	25.83 ± 1.30 nM	0.9202 (ns)
		EGFP	27.03 ± 1.83 nM	0.4528 (ns)
Figure 3D<br>Supplementary Figure 4B	14c	3D7	2.42 ± 0.07 μM	/
		HO	8.41 ± 1.84 μM	0.0049 (**)
		EGFP	2.38 ± 0.24 μM	0.7954 (ns)
Figure 3F	HO(ART)	Control	18.37 ± 0.76 nM	/
		14c	17.47 ± 1.03 nM	0.2902 (ns)

Table S1D. Heme analogues-ZnPPIX/ZnMP increased EC_{50} of artemisinin.

Sample		EC_{50} Value (nM)	*p*-Value (*t*-test)
Figure 4B	Control	25.61 ± 2.32	/
	5 μM ZnPPIX	40.96 ± 2.50	0.0015 (**)
	10 μM ZnPPIX	69.02 ± 6.96	0.0005 (***)
Figure 4D	Control	25.61 ± 2.32	/
	5 μM ZnMP	39.49 ± 2.88	0.0029 (**)
	10 μM ZnMP	79.27 ± 5.29	<0.0001 (***)

Table S1E. Inhibitors of heme biosynthesis and hemoglobin degradation had little effect on the efficacy of artemisinin.

Sample		EC_{50} Value (nM)	*p*-Value (*t*-test)
Figure 5C	Control (H_2O)	23.32 ± 1.28	/
	50 μM SA	22.96 ± 1.16	0.7324 (ns)
	100 μM SA	21.94 ± 1.17	0.2402 (ns)
Figure 5F	Control (DMSO)	26.97 ± 1.10	/
	5 μM E64	25.00 ± 1.37	0.1241 (ns)
	10 μM E64	25.32 ± 1.08	0.1284 (ns)
	80 μM Pepstatin A	24.48 ± 1.18	0.0556 (ns)
	160 μM Pepstatin A	27.87 ± 4.22	0.7388 (ns)
Figure 5H	Control (DMSO + H_2O)	31.22 ± 5.56	/
	E64 + Pepstatin A	22.68 ± 3.22	0.0827 (ns)
	E64 + SA	24.56 ± 3.34	0.1503 (ns)
	Pepstatin A + SA	25.26 ± 1.62	0.1491 (ns)
	E64 + Pepstatin A + SA	22.59 ± 3.22	0.0806 (ns)

Supplementary Table S2. EC_{50} values of some antimalarials with heme related chemicals.

Table S2A. Elevating heme level could not affect atovaquone action.

Sample		EC_{50} Value (nM)	*p*-Value (*t*-test)
Figure 6A	Control (0.1M NaOH)	0.61 ± 0.08	/
	5 µM Hemin	0.66 ± 0.09	0.5118 (ns)
	10 µM Hemin	0.61 ± 0.09	>0.9999 (ns)
Figure 6B	Control (DMSO)	0.74 ± 0.13	/
	5 µM ZnMP	0.60 ± 0.07	0.1759 (ns)
	10 µM ZnMP	0.60 ± 0.08	0.1874 (ns)
Figure 6C	Control (DMSO)	0.74 ± 0.13	/
	5 µM ZnPPIX	0.65 ± 0.08	0.3649 (ns)
	10 µM ZnPPIX	0.68 ± 0.08	0.5334 (ns)
Figure 6D	Control (DMSO)	0.74 ± 0.13	/
	3 µM 14c	0.75 ± 0.10	0.9210 (ns)
	5 µM 14c	0.65 ± 0.10	0.3957 (ns)

Table S2B. Hemin exerted little effect on some other antimalarials.

Sample		Control	Hemin	*p*-Value (*t*-test)
Figure 6E	Artemisinin	20.57 ± 1.22 nM	>200 nM	/
Figure 6F	Quinine	115.05 ± 11.55 nM	130.35 ± 14.15 nM	0.2180 (ns)
Figure 6G	Mefloquine	44.21 ± 3.44 nM	38.66 ± 2.43 nM	0.0846 (ns)
Figure 6H	Proguanil	22.41 ± 1.55 µM	22.27 ± 1.4 µM	0.9132 (ns)
Figure 6I	Pyrimethamine	85.52 ± 18.59 nM	75.35 ± 14.88 nM	0.5045 (ns)

Supplementary Figures & Legends

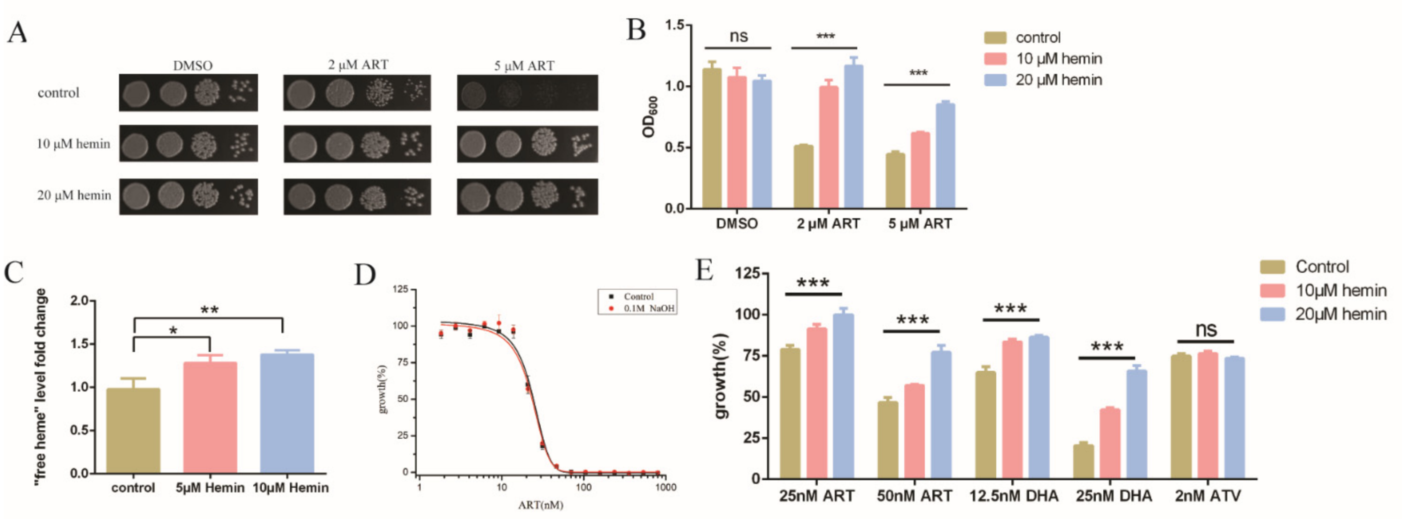

Figure S1. Heme antagonized artemisinins' action. Sensitivity of artemisinin in *Saccharomyces cerevisiae* was decreased with the addition of hemin on agar plates (**A**) and in liquid (**B**). For liquid assays, data are mean ± SD of four independent experiments. Comparison was performed by the analysis of variance (ANOVA), *** $p < 0.0005$, ns: not significant ($p > 0.05$). (**C**) Exogenous hemin enhanced "free heme" level in parasites. Data are mean ± SD of three independent experiments, comparison was performed by the unpaired t-test, * $p < 0.05$, ** $p < 0.005$. (**D**) 0.1 M NaOH had little effect on artemisinin action. Data are mean ± SD of three independent experiments, $p = 0.8547$ (not significant). (**E**) Pre-treated with hemin decreased ARTs efficacy. Prior treatment of parasites with hemin for 24 h followed by washing, short pulse assay at middle ring stage was used for artemisinins sensitivity measurement, atovaquone (ATV) as a control. Data are mean ± SD of three independent experiments. Comparison was performed by the analysis of variance (ANOVA), *** $p < 0.0005$, ns: not significant ($p > 0.05$).

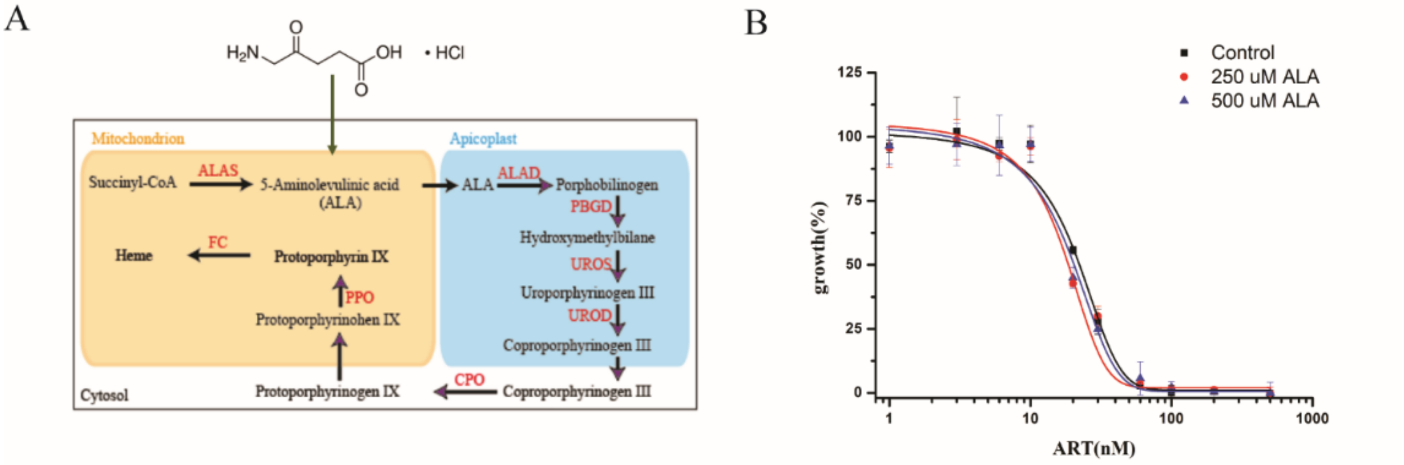

Figure S2. ALA did not affect artemisinin potency. (**A**) ALA is a precursor of heme biosynthesis. (**B**) Sensitivity of artemisinin with ALA had no significant change compared with H_2O. EC_{50} values were 20.02 ± 0.22 nM/19.83 ± 0.24 nM/19.87 ± 0.22 nM in H_2O/250 μM ALA/500 μM ALA. Data are mean ± SD of three independent experiments, $p > 0.05$ (not significant).

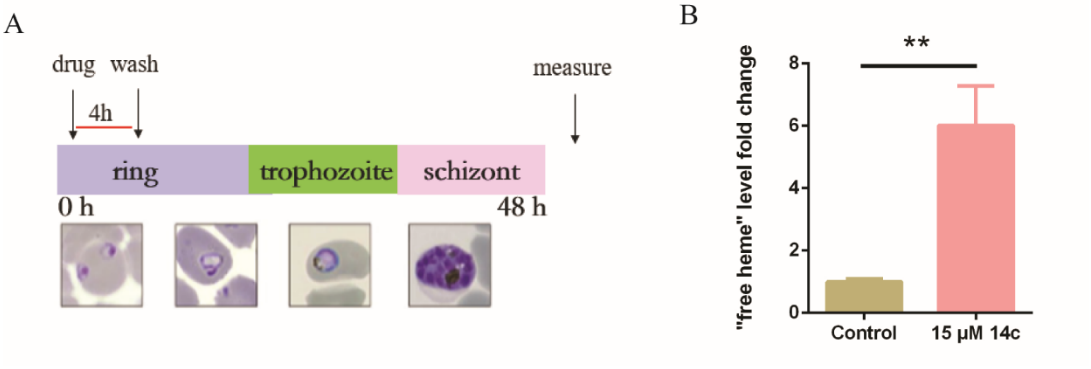

Figure S3. Short pulse assay of 14c. (**A**) A schematic representation of the short pulse assay. (**B**) The free heme level was enhanced with 15 μM 14C after 4 h incubation. Data are mean ± SD of three independent experiments, ** $p < 0.005$.

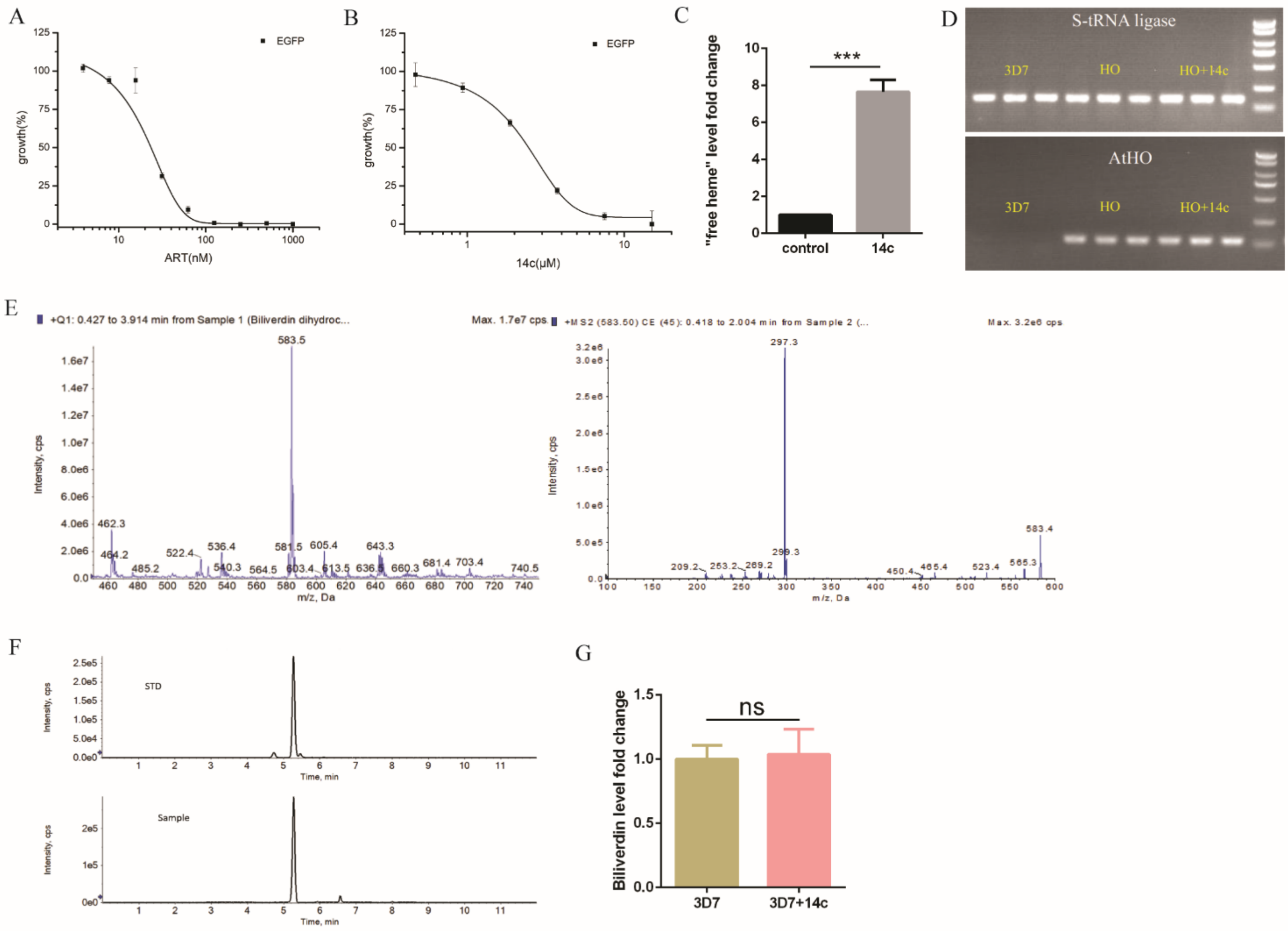

Figure S4. HO was functional in the transgenic parasite. HO gene was introduced to the parasites. EGFP expression strain was used as a control. (**A**) and (**B**) are respectively sensitivity to artemisinin (**A**) and to 14c (**B**) of the EGFP expression strain. EC_{50} values are shown in Supplementary Table S1C. (**C**) "free heme" level was enhanced by 14c in the EGFP expression strain. Data are mean ± SD of three independent experiments. *** $p < 0.0005$. (**D**) HO expression of the transfected clones was confirmed by RT-qPCR, with serine-tRNA ligase gene as the control. (**E**) Expanded y axis views of the peaks of biliverdin standard sample detected by LS-MS/MS. (**F**) Comparison of standard (STD) sample and experimental sample. (**G**) Biliverdin level in 3D7 infected RBC had little change with 14c. Data are mean ± SD of four independent experiments. ns: not significant ($p > 0.05$).

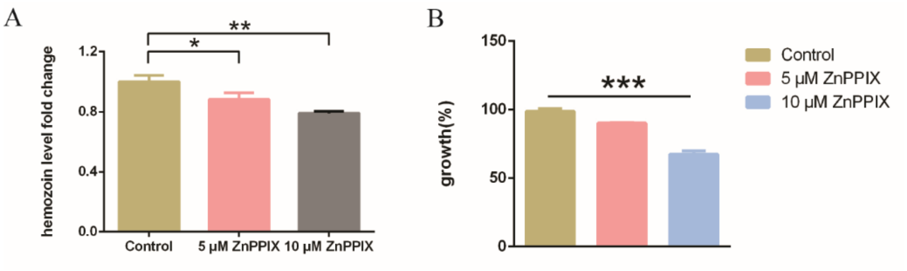

Figure S5. ZnPPIX had effect on hemozoin formation and parasite growth. (**A**) ZnPPIX reduced hemozoin level in parasites. Data are mean ± SD of three independent experiments, * $p < 0.05$, ** $p < 0.005$. (**B**) ZnPPIX could inhibit the growth of *p. falciparum*. The growth of parasites was measured after 72 h with ZnPPIX. Data are mean ± SD of three independent experiments, comparison was performed by the analysis of variance (ANOVA), *** $p < 0.0005$.

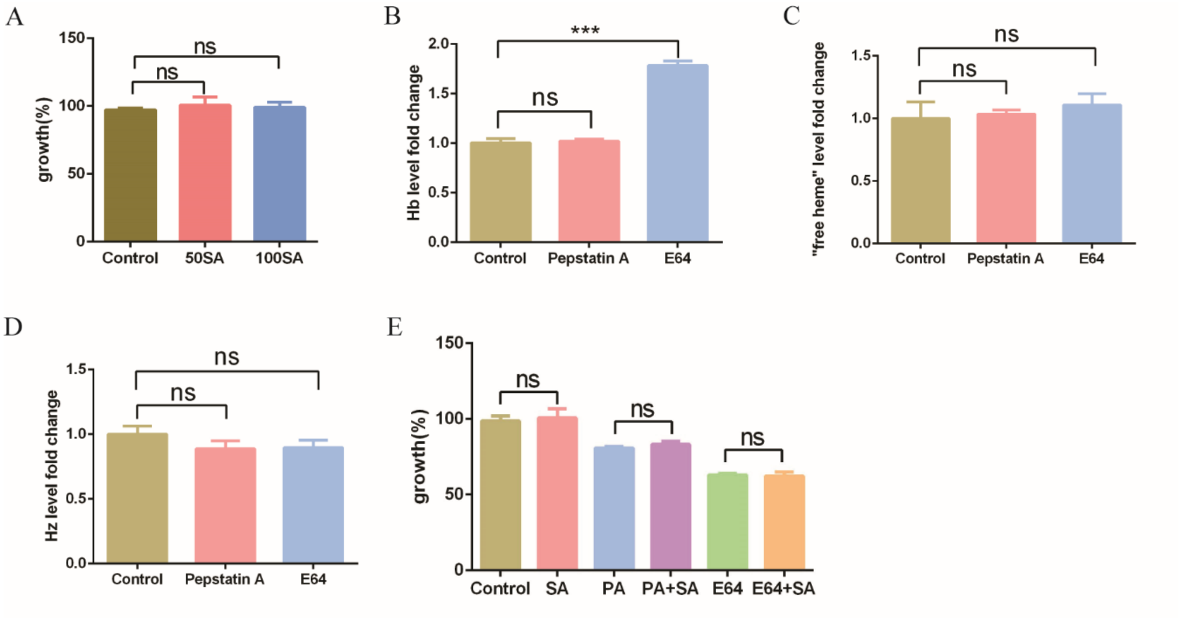

Figure S6. Inhibitors of heme related pathway had little effect on parasite growth or free heme level. (**A**) 50 μM or 100 μM SA had no toxic effect on parasites. The growth of parasites was measured after 72 h with SA. Data are mean ± SD of three independent experiments, ns: not significant ($p > 0.05$). E64 inhibited heme degradation but had little effect on "free heme" level and hemozoin level. (**B**) Hemoglobin level in parasite in the presence of inhibitors-E64 and Pepstatin A. "free heme" level and hemozoin level with E64 and Pepstatin A are shown in (**C**) and (**D**), respectively. Data are mean ± SD of three independent experiments, ns: not significant ($p > 0.05$), *** $p < 0.0005$. (**E**) SA had no interaction with E64 or Pepstatin A. The growth of parasites was measured after 72 h with heme related inhibitors. Data are mean ± SD of three independent experiments, ns: not significant ($p > 0.05$).
